# Mechanisms and Research Progress of SGLT-2 Inhibitors in Diabetes Mellitus-Related Cognitive Impairment

**DOI:** 10.2174/0115733998444053260211052025

**Published:** 2026-03-23

**Authors:** Yutong Li, Murong Xu, Ying Tang, Xiaotong Zhao, Mingwei Chen

**Affiliations:** 1 Department of Endocrinology, The First Affiliated Hospital of Anhui Medical University, Hefei, Anhui, 230032, P.R. China

**Keywords:** Sodium-glucose cotransporter 2 inhibitors, diabetes mellitus-related cognitive impairment, diabetes mellitus, mild cognitive impairment, dementia, Alzheimer's disease

## Abstract

Diabetes Mellitus (DM) increases the risk of Mild Cognitive Impairment (MCI) and dementia, severely impacting patients' quality of life and long-term future prospects. Sodium-glucose cotransporter 2 inhibitors (SGLT-2i), a class of oral hypoglycemic drugs, have recently been shown to have neuroprotective effects. Studies show that SGLT-2i ameliorates cognitive dysfunction in diabetic animal models through various mechanisms such as insulin pathways in the brain, cerebrovascular dysfuntion, neuroinflammation and oxidative stress, inhibition of Alzheimer's disease pathology, and neurotrophic factor expression. Clinical studies also show that SGLT-2i improves cognitive performance in diabetic patients, significantly reducing the risk of dementia and MCI. Despite the promising role of SGLT-2i in preventing and treating DM-related cognitive impairment, further studies and more robust clinical evidence are needed for its clinical application.

## 1 INTRODUCTION

Diabetes Mellitus (DM) refers to a metabolic disorder characterized by the chronic elevation of blood glucose due to inadequate insulin production or impaired insulin utilization. As the population ages and lifestyle changes, the global prevalence of Diabetes Mellitus (DM) has increased rapidly, and it is a major public health problem worldwide [1]. Recently, cognitive dysfunction has become a notable complication associated with DM [2]. Cognitive impairment is classified into Mild Cognitive Impairment (MCI) and dementia. In China, MCI and dementia prevalence rates are approximately 15.5% and 6.0% among elderly people over 60 years old, respectively [3]. DM also increases the risk of MCI and dementia by 1.44- and 2.14-fold, respectively, compared to individuals without DM [3]. In a longitudinal cohort study in the United States, MCI prevalence among newly diagnosed patients with DM was 42.9% over ten years, corresponding to a 1.5-fold higher risk relative to individuals without a DM diagnosis [4].

Although the specific mechanisms of DM-related cognitive impairment are not known, many studies have suggested several mechanisms are involved, including neural and vascular damage caused by DM, Insulin Resistance (IR) with dysregulated insulin signaling, neuronal damage due to hypoglycemia, chronic brain inflammation, microvascular dysfunction in the cerebral circulation, and accumulation of amyloid and tau protein aggregates in neural tissues [2, 5, 6]. Patients with DM often have impairments across multiple cognitive domains, including attention, language skills, visual memory, executive functioning, motor coordination, and information processing speed [7]. These impairments severely affect patients' self-care and impede their ability to perform daily tasks. Early detection, prevention, and treatment of DM-related cognitive impairments are important to improve patient prognosis.

SGLT-2 is usually located on the surface of the apical membrane of epithelial cells within renal proximal tubules. It plays a role in active reabsorption of glucose from renal filtrate through sodium-glucose cotransport. Pharmacologically, sodium-glucose cotransporter 2 inhibitors (SGLT-2i) specifically interrupt this transport, promoting glucose excretion through urine and reducing circulating glucose levels. Dapagliflozin was first clinically available as an SGLT-2i in Europe in 2012. Several other SGLT-2i, such as empagliflozin, canagliflozin, and ertugliflozin, were developed to expand available therapeutic options and individualized treatment for patients with DM. Numerous large-scale clinical trials evaluating cardiovascular outcomes in diabetic populations have consistently shown positive effects of SGLT-2i on cardiovascular and renal endpoints. Cardiovascular benefits of SGLT-2i have been extended to non-DM patients with heart failure with reduced ejection fraction (HFrEF) [8]. Recently, increasing attention has focused on the neuroprotective effects of SGLT-2i. SGLT-2i has demonstrated significant clinical potential in improving cognitive function and preventing cognitive impairment in type 2 DM patients [9]. We searched the Medline, Cochrane, and Embase databases for relevant literature up to June 2025 using three groups of keywords: diabetes, SGLT-2 inhibitors, and cognitive impairment. Studies that investigated molecular mechanisms and clinical effects of SGLT-2i in DM-related cognitive impairment were ultimately included. This review summarizes the clinical evidence and potential mechanisms through which SGLT-2i improve DM-related cognitive impairment and discusses potential applications.

### 1.1 Search Strategy

 We systematically searched the Medline, Cochrane, and Embase databases for relevant literature published up to June 2025. The search was conducted using three groups of keywords and their combinations: "diabetes", "SGLT-2 inhibitors", and "cognitive impairment".

Inclusion criteria: Studies investigating the molecular mechanisms and clinical effects of SGLT-2i in diabetes-related cognitive impairment were ultimately included. 

Exclusion criteria: Studies mentioning keywords but whose core content is unrelated to SGLT-2i’s role in cognitive impairment were excluded. Editorials, letters, meeting abstracts, or non-peer-reviewed grey literature were excluded. Studies with significant defects in experimental design, poor data quality, or inappropriate statistical analysis were also excluded.

## 2 PATHOLOGICAL MECHANISMS OF DM-RELATED COGNITIVE IMPAIRMENT

Cognition is the complex, advanced functions of the cerebral cortex. Cognitive impairment arises from many pathological factors that damage nerves and affect synaptic plasticity. Cognitive impairment is commonly classified into dementia and MCI, depending on severity. Alzheimer's disease, cardiovascular disease, Lewy body disease, and frontotemporal dementia, characterized by severe deficits in many cognitive domains, are considered dementias. MCI is the transition between normal cognitive aging and dementia. Although no precise link between DM and cognitive impairment is known yet, current research suggests that it primarily involves the following aspects:

### 2.1 Brain IR and Impaired Insulin Signaling

Insulin receptors are widely distributed in both presynaptic and postsynaptic neurons [10]. After binding to insulin receptors, insulin regulates synaptic function through the PI3K-Akt pathway [11]. This regulation involves: promoting the transport of Glucose Transporter 4 (GLUT4) to the neuronal plasma membrane for glucose uptake [12], phosphorylating the AMPA-type glutamate receptor subunit GluA1 and increasing synaptic localization of the NMDA receptor subunit GluN2B to improve synaptic plasticity and memory [13], activating the mTOR pathway for protein and lipid production, which promotes dendritic spine formation and neuronal development [14], and phosphorylating GSK3 at Ser9 to inhibit its activity, since abnormally activated GSK3 can lead to hyperphosphorylation of tau protein [15]. The IR and hyperinsulinemia in Type 2 Diabetes Mellitus (T2DM) disrupt insulin signaling pathways, affecting neuronal energy metabolism, protein homeostasis, and vascular function, and hence neurodegeneration [16].

### 2.2 Vascular Injury and Circulatory Dysfunction

Recent evidence shows a strong connection between cognitive impairment and cerebrovascular disorders [17]. Cerebral Small Vessel Disease (SVD) plays a key role in cognitive dysfunction [18, 19]. Chronic hyperglycemia promotes the accumulation of toxic lipids, Advanced Glycation End products (AGEs), and amyloid deposits in vascular walls. Sustained hyperglycemia stimulates Reactive Oxygen Species (ROS) generation, decreases production of vasodilatory agents, impairs endothelial cell function, slows cerebral blood flow, and ultimately contributes to neuronal injury [20]. Imaging studies have shown that WMH volume correlates with slower information processing speed in chronic poor glycemic control patients with T2DM, suggesting cerebral SVD as a cognitive impairment mechanism [21]. In addition, DM increases the risk of major arterial stenosis and occlusion. It has been shown that lack of cerebral blood flow due to carotid artery stenosis or occlusion directly leads to cognitive decline [22]. A study using Arterial Spin Labeling (ASL) Magnetic Resonance Imaging (MRI) combined with neuropsychological assessments showed that decreased cerebral perfusion in T2DM patients closely correlated with cognitive impairment [23].

### 2.3 Neuroinflammation and Oxidative Stress (OS)

Increased expression of pro-inflammatory cytokines, including Tumor Necrosis Factor (TNF), Interleukin (IL)-1β, IL-2, and IL-6, has been observed in the brains of patients with DM, activating microglia and triggering neuroinflammatory cascades [24]. Animal models support this relationship: diabetic obese (db/db) mice, commonly used as a metabolic syndrome model, had impaired spatial recognition memory associated with increased hippocampal levels of pro-inflammatory cytokines (TNF-α, IL-1β, IL-6) and decreased expression of Brain-Derived Neurotrophic Factor (BDNF) [25]. Disturbances in glucose and lipid metabolism increase ROS production, resulting in elevated OS, which leads to mitochondrial dysfunction, impairing energy supply and consequently causing neuronal damage and neurodegeneration [26].

### 2.4 Pathological Overlap Between DM and AD

There is obvious overlap in the pathologies of diabetes and AD. The abnormal aggregation and deposition of amyloid-beta (Aβ) proteins is one of the key pathological features of AD. Both Aβ and insulin are substrates of the Insulin-Degrading Enzyme (IDE). Increased insulin levels in the brain inhibit IDE activity and thereby decrease clearance of Aβ [27]. Chronic hyperglycemia promotes β-secretase 1 (BACE1) activity and thereby accelerates Aβ production and accumulation [28]. Amylin, co-secreted by pancreatic beta cells along with insulin, accumulates abnormally in the brains of T2DM and AD patients. Amylin binds to Aβ, forming neurotoxic mixed plaques and contributing to cognitive impairment [29].

### 2.5 Dysglycemia and Excessive HPA Axis Activation

Hypoglycemia is a common problem for insulin-treated patients with DM treated with insulin. Severe hypoglycemia can cause selective neuronal death and cognitive impairment [30]. Chronic hyperglycemia and IR activate the Hypothalamic-Pituitary-Adrenal (HPA) axis, resulting in excessive cortisol secretion. Recent studies have shown increased cortisol levels in the cerebrospinal fluid of MCI patients [31]. Increased cortisol may further exacerbate AD pathology through mechanisms such as enhanced Aβ accumulation, heightened tau hyperphosphorylation, and aggravated OS [32].

## 3 MECHANISMS OF SGLT-2I IN IMPROVING DM-RELATED COGNITIVE IMPAIRMENT

The SGLT family includes two main isoforms: SGLT-1 and SGLT-2. SGLT-1 is most abundantly expressed in the small intestine and renal proximal tubular cells, whereas SGLT-2 is most abundantly expressed in renal proximal tubules. Both SGLT isoforms are also expressed differently in the Central Nervous System (CNS). SGLT-1 has been identified predominantly in pyramidal cells of the cerebral cortex, Purkinje neurons of the cerebellum, and both pyramidal and granular neurons in the hippocampus [33]. SGLT-2 exhibits lower CNS expression, mainly concentrated in cerebral microvascular regions of the blood-brain barrier, the hypothalamus, the amygdala, the periaqueductal gray matter, and the dorsal medulla [33]. The cerebral distribution patterns of these transporters are closely linked to cognition. Accumulating evidence indicates that SGLT-2i exerts neuroprotective effects and ameliorates cognitive impairment *via* multiple pathways (Fig. **1**).

### 3.1 Regulation of Brain Insulin Signaling and Glucose Metabolism

Abnormalities in brain insulin signaling play important roles in DM-related cognitive impairments. According to previous animal studies, rats with insulin resistance induced by a High-Fat Diet (HFD) displayed notable cognitive deficits [34]. Dapagliflozin treatment significantly improved insulin-mediated Long-Term Depression (LTD) and increased insulin receptor tyrosine phosphorylation and serine phosphorylation of Akt in the brain [34]. These observations indicate that dapagliflozin can restore insulin sensitivity and improve impaired insulin signaling in rats with insulin resistance induced by an HFD [34]. The mTOR signaling pathway, involved in regulating metabolism, cell growth, and protein synthesis, represents a potential therapeutic target for DM-related cognitive impairment. In an aluminum chloride-induced AD rat model, dapagliflozin administration dose-dependently enhanced spatial memory and learning through activation of the AMPK and mTOR signaling pathways. Additionally, this treatment reduced cerebral glucose levels while upregulating glucose transporters (GLUT-1 and GLUT-3) and key glycolytic enzymes, including Lactate Dehydrogenase A (LDH-A) and pyruvate dehydrogenase kinase 1 (PDK-1) [35]. Conversely, in a doxorubicin (DOX)-induced chemotherapy brain injury model in rats, empagliflozin alleviated cognitive impairment by inhibiting excessive activation of the PI3K/pAkt/mTOR axis and suppressing downstream inflammatory cytokines [36]. Another study reported that canagliflozin improved central insulin sensitivity and reduced hippocampal mTOR signaling in elderly mice, exhibiting sex-specific effects [37]. Thus, SGLT-2i may have differential effects on the mTOR pathway depending on animal models, drug types, and dosages [38]. In the aluminum chloride-induced rat model of AD, inhibition of the PI3K/Akt/mTOR pathway promotes neurodegeneration and subsequently leads to cognitive impairment. In contrast, in the rat model of chemobrain, hyperactivation of the PI3K/p-Akt/mTOR axis contributes to cognitive deficits by exacerbating neuroinflammation and oxidative stress. Both inhibition and excessive activation of the mTOR pathway can induce cognitive impairments, but SGLT-2i ultimately exerts a protective effect on cognitive function. Moreover, Chronic Social Defeat (CSD) induces pronounced hyperglycemia in mice, leading to high glucose levels in the hippocampus and thereby results in spatial memory impairments [39]. Blood glucose reduction with empagliflozin reverses glucose metabolic disturbances and spatial memory deficits in CSD-induced hyperglycemia-sensitive mice [39]. This research shows the potential of SGLT-2i in treating comorbid metabolic and cognitive disorders associated with chronic psychosocial stress.

### 3.2 Improvement of Cerebrovascular Dysfunction

Cerebrovascular pathology significantly influences cognitive deficits in patients with DM. Studies indicate that impaired Myogenic Responses (MR) in middle cerebral arteries and parenchymal arterioles compromise cerebral blood flow autoregulation in diabetic rat models. Concurrent disruption of Blood-Brain Barrier (BBB) integrity, tight junction abnormalities, decreased pericyte coverage, and diminished microvascular networks in the cerebral cortex and hippocampal CA3 regions further deteriorate short-term memory function [40]. Treatment using luseogliflozin effectively restored MR function, improved cerebral blood flow regulation, enhanced BBB integrity, and increased neuronal density within the hippocampal CA3 area, consequently improving short-term cognition in diabetic rats [41]. Moreover, luseogliflozin improved the contractility of Vascular Smooth Muscle Cells (VSMCs) under high-glucose conditions *in vitro* [41]. Additionally, SGLT-2 inhibitors have shown protective effects against cerebral Ischemia/Reperfusion (I/R) injury [42, 43]. Empagliflozin significantly reduced infarct size and improved neurobehavioral outcomes in a global cerebral I/R injury rat model in a dose-dependent manner. Its neuroprotective mechanism involved attenuation of neuronal apoptosis *via* the HIF-1α/VEGF signaling pathway [42]. Hypertension, another significant risk factor for cerebrovascular disorders, is effectively mitigated by SGLT-2 inhibitors through osmotic diuresis-induced sodium excretion, suppression of the renin-angiotensin system activation, reduction of sympathetic nervous system activity, enhanced antioxidative defenses, and improvement of endothelial cell function [44].

### 3.3 Attenuation of Neuroinflammation

Neuroinflammation is a critical pathological factor contributing to cognitive impairment. Microglia, the primary immune cells of the CNS, become excessively activated under pathological conditions, polarizing toward a pro-inflammatory phenotype (M1), releasing inflammatory cytokines, and generating ROS, which exacerbate neuroinflammation. Numerous studies indicate that SGLT-2i inhibits microglial activation and polarization, thereby reducing neuroinflammation [37, 45, 46]. An *in vitro* study using BV-2 microglia cultured under high-glucose conditions showed that canagliflozin was more effective than dapagliflozin, empagliflozin, and ertugliflozin in suppressing high-glucose-induced OS, inflammatory cytokine release, apoptosis, and autophagy-related signaling [46]. Additionally, abnormal Neurovascular Unit (NVU) remodeling occurred in T2DM female mice, characterized by disrupted endothelial tight junctions, pericyte loss, thickened basement membranes, activated astrocytes detaching from vessel walls, and microglial activation with mitochondrial dysfunction. Empagliflozin reversed NVU remodeling, reducing neuroinflammation and neuronal injury [45]. In spontaneous epilepsy mouse models, dapagliflozin significantly reduced seizure frequency and improved cognitive function by suppressing microglial activation and reducing IL-6, IL-18, and IL-1β production [47]. Furthermore, SGLT-2i reduces neuroinflammation by inhibiting NF-κB and downstream pathways [48-50]. Canagliflozin has been shown to alleviate cognitive deficits induced by methotrexate in rat models by suppressing the TLR4/NF-κB/NLRP3 signaling cascade and facilitating microglial polarization toward the anti-inflammatory M2 phenotype, thereby restoring the equilibrium of microglial phenotypic states [50].

### 3.4 Reduction of OS and Mitochondrial Dysfunction

OS, resulting from excessive ROS accumulation, induces mitochondrial dysfunction and accelerates neurodegeneration, leading to cognitive impairment. Common OS markers include indicators of oxidative damage and antioxidants. Multiple animal studies show that SGLT-2i reduces ROS production, decreases MDA and 8-OHdG levels, and raises GSH, SOD, and CAT activities, thus improving cognitive outcomes [35, 36, 41, 51, 52]. Mitochondria are essential for cell energy production and function. *In vitro* studies show empagliflozin reduces mitochondrial Ca^2+^ overload and reduces mitochondrial ROS production in high-glucose-treated endothelial cells, alleviating mitochondrial OS [53]. In a mouse model of DM obtained by streptozotocin induction, dapagliflozin enhanced spatial learning and memory capabilities. Histopathological analysis revealed structural and organizational recovery of neurons in the hippocampal CA1, CA3, and dentate gyrus regions. Dapagliflozin prevented DM-related disruption of mitochondrial outer membrane integrity, promoted mitochondrial fusion by increased expression of related fusion proteins, and reversed the abnormalities observed in mitochondrial fission [54].

### 3.5 Effects of SGLT-2i on AD Pathology

T2DM and AD share common pathological features, notably the abnormal aggregation and deposition of Aβ and hyperphosphorylation of tau protein. Growing evidence suggests that SGLT-2i can reduce the abnormal deposition of Aβ and excessive phosphorylation of tau. In an aluminum chloride (AlCl_3_)-induced AD rat model, dapagliflozin significantly inhibited AlCl_3_-induced elevation of acetylcholinesterase (AChE) activity and Aβ levels in a dose-dependent manner [35]. Elevated AChE activity accelerates acetylcholine (ACh) degradation, impairing cholinergic neurotransmission and promoting abnormal Aβ deposition [55]. In a streptozotocin (STZ)-induced sporadic AD (SAD) mouse model, canagliflozin inhibited the hippocampal expression of tau, AChE, BACE1, and Aβ, while promoting IDE expression [49]. Reduced BACE1 expression decreases Aβ deposition, while increased IDE enhances Aβ clearance. Another study found that empagliflozin significantly improved learning and memory in mouse models with AD, T2DM, or combined AD-T2DM pathology [56]. Empagliflozin also reduced cortical brain atrophy, senile plaque (SP) burden, cortical Aβ40 levels, and tau phosphorylation in AD-T2DM mice [56]. Furthermore, in a mouse model of HFD-induced cognitive impairment, dapagliflozin increased insulin receptor expression in hippocampal tissue and reduced phosphorylated tau levels [57].

### 3.6 Regulation of Neurotrophic Factors by SGLT-2i

Neurotrophic factors, small proteins secreted predominantly by neuronal and glial cells, play essential roles in regulating neuronal survival, growth, and synaptic functionality. Prominent members of this group include BDNF, Nerve Growth Factor (NGF), Glial-Derived Neurotrophic Factor (GDNF), and neurotrophin-3 and -4 (NT-3/NT-4), whose expression levels are typically altered in cognitive impairment conditions. Previous studies have demonstrated that empagliflozin and dapagliflozin enhanced cognitive function in diabetic mouse models by significantly elevating BDNF protein expression within the hippocampus and prefrontal cortex. Furthermore, empagliflozin treatment selectively increased NT-4 levels in the prefrontal cortex [58, 59]. Another investigation revealed that canagliflozin markedly raised BDNF levels in the hippocampus of STZ-induced SAD mouse models [49]. Additionally, empagliflozin administration in a rotenone-induced Parkinson’s Disease (PD) animal model enhanced neural plasticity by upregulating cerebral expression of BDNF, cAMP response element-binding protein (CREB), and neuronal PAS domain protein 4 (Npas4) [59]. In a fisetin-triggered PD model, dapagliflozin reversed the inhibitory impact of fisetin on the PI3K/AKT/GSK-3β pathway through increased GDNF expression in striatal regions, thus exerting neuroprotective, anti-apoptotic effects [48].

## 4 CLINICAL RESEARCH ON SGLT-2I FOR DM-RELATED COGNITIVE IMPAIRMENT

Since the mechanisms of SGLT-2i-mediated cognitive improvement have become more and more important, its clinical relevance has attracted considerable attention. Clinical studies have investigated the effects of SGLT-2i on DM-related cognitive impairment. Evaluations typically employ neuropsychological assessment scales, biomarkers, or brain imaging. Neuropsychological assessment tools, including the Mini-Mental State Examination (MMSE) and the Montreal Cognitive Assessment (MoCA), are used to quantify cognitive function, emotional state, behavioral performance, and overall brain functional integrity. Biomarker analyses involve cerebrospinal fluid levels of Aβ42, total tau protein, and phosphorylated tau at threonine 181 (p-tau181). Brain imaging studies use MRI to visualize cerebral vascular, brain atrophy, and cerebral perfusion. Based on these methods, many clinical studies have shown the beneficial effects of SGLT-2i on DM-related cognitive impairment. Table **1** summarizes key clinical studies published in recent years, including their study designs and primary outcomes.

The clinical evidence presented in Table **1** indicates that SGLT-2i, including dapagliflozin, canagliflozin, empagliflozin, ertugliflozin, and henagliflozin, improve cognitive function in patients with DM [60-67] and lower the risk of dementia and MCI [60, 61, 67-73]. A meta-analysis suggested that SGLT-2i reduces dementia risk by about 16% in patients with T2DM [60]. Results from cohort studies and Randomized Controlled Trials (RCTs) suggest that SGLT-2i improves cognitive performance and reduces the risk of cognitive impairment in patients with DM-related conditions such as heart failure [66, 67], Chronic Kidney Disease (CKD) [62], and atrial fibrillation [71]. Retrospective studies directly comparing SGLT-2 inhibitors with dipeptidyl peptidase-4 inhibitors (DPP-4i) showed that dementia is more protected by SGLT-2i [68, 70, 72]. A Canadian retrospective cohort study in Ontario found that dapagliflozin had superior efficacy in reducing dementia incidence compared with other SGLT-2 inhibitors [68]. A clinical trial of henagliflozin, a novel SGLT-2 inhibitor, showed significantly lower plasma p-tau181 levels and improved cognitive function in patients with T2DM cognitive deficits [65]. Clinical studies also showed many ancillary benefits of SGLT-2i, including enhanced daily living activities [62, 63, 66], improved self-management efficacy [63], reduced depressive symptoms [66], and decreased risk of Parkinson’s disease [72]. Despite generally positive findings, some inconsistencies remain. One RCT on elderly patients with T2DM reported no significant cognitive improvement following 12 months of SGLT-2i treatment [75]. Current clinical research also has limitations, such as insufficient systematic reviews or meta-analyses on cognitive improvements from SGLT-2i, making it difficult to assess heterogeneity and overall effect sizes across studies. High-quality multicenter RCTs and prospective cohort studies are scarce. Evaluation tools, study populations, intervention durations, and follow-up periods vary greatly between studies, which is why more robust long-term data are needed.

## 5 DISCUSSION, SUMMARY, AND OUTLOOK

DM-related cognitive impairment is a common complication of diabetes that affects patients’ QoL and long-term prognosis. SGLT-2i, a multifunctional oral hypoglycemic drug, has been gaining attention for its neuroprotective properties. This review summarizes preclinical mechanistic studies and relevant clinical research on SGLT-2 inhibitors and compares them with other glucose-lowering agents to preliminarily explore their relative neuroprotective advantages. By summarizing current data, we hope to provide a reference for understanding the potential role of SGLT-2 inhibitors in the prevention and treatment of DM-related cognitive impairment.

Basic research indicates that SGLT-2i induced neuroprotective effects and improved cognitive function in animal models of DM-related cognitive impairment through various mechanisms, including regulation of cerebral insulin signaling and glucose metabolism, reduction of cerebrovascular dysfunction, reduction of neuroinflammation, attenuation of OS, improvement of mitochondrial function, regulation of AD pathology, and regulation of neurotrophic factor expression. These mechanistic studies provide important hypotheses and evidence for potential novel applications of SGLT2 inhibitors.

## 6 STUDY LIMITATIONS

However, several limitations remain. First, the studies are based on heterogeneous animal models, and none of the models can fully recapitulate the natural course of cognitive impairment-related disorders in humans. Second, the behavioral tests in animals only evaluate limited aspects of learning and memory, fundamentally different from the complex and multidimensional cognitive functions in humans. This introduces significant uncertainty when extrapolating “cognitive improvement” to clinical settings. Finally, the pathological mechanisms of DM-related cognitive impairment are highly interconnected rather than isolated. Most existing studies examine single pathways in relative isolation, while SGLT-2i are more likely to act through multiple, interrelated signaling pathways simultaneously. Together, the mechanisms of cognitive benefits of SGLT-2 inhibitors are incompletely described and deserve further clarification through comprehensive *in vitro* and animal model studies.

Clinical results show that SGLT-2 inhibitors can help improve cognitive performance and lower the risk of cognitive impairment among diabetic individuals with related comorbidities. Current clinical investigations are limited by small sample sizes, brief follow-up periods, and variability in cognitive evaluation standards. Most studies lack neuroimaging data to corroborate the observed cognitive effects with corresponding changes in brain structure and function. Future studies should address these limitations to elucidate the neuroprotective potential of SGLT-2i. Future research should also involve larger-scale, higher-quality clinical trials with longer follow-up periods and systematic meta-analyses of available data.

## CONCLUSION

Current evidence suggests that SGLT-2i may have clinical value and potential in the prevention and management of DM-related cognitive impairment. However, the current evidence remains insufficient to fully establish its clinical application paradigm. Future work should be directed toward further elucidating its mechanisms of action and validating its efficacy through more rigorous, prospective clinical studies of higher evidence levels.

## Figures and Tables

**Fig. (1):**
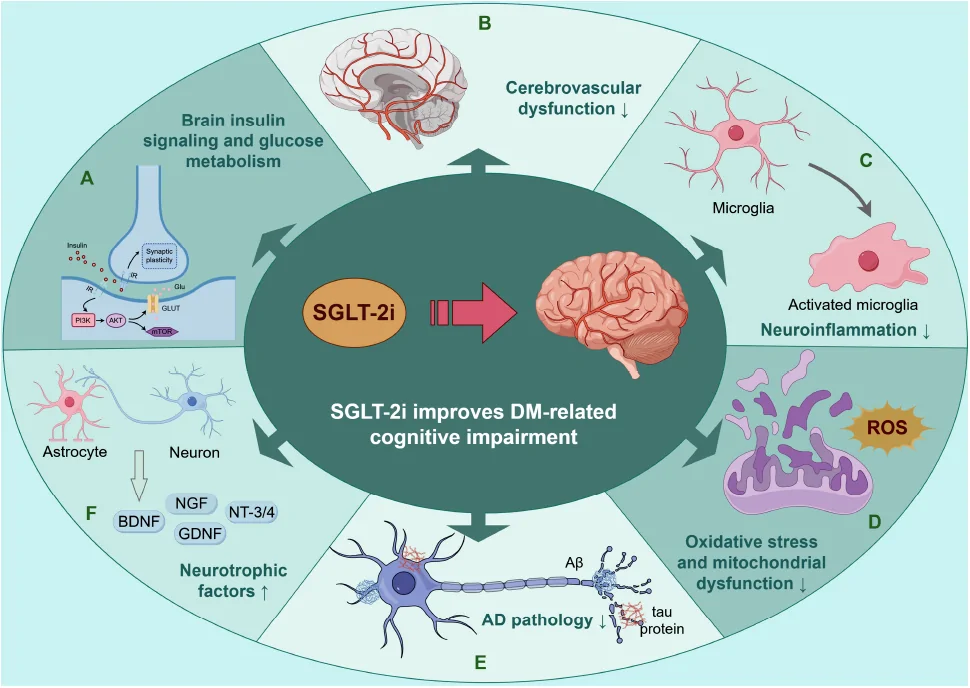
Mechanisms of SGLT-2i in improving DM-related cognitive impairment. ↑: increase; ↓: decrease. (**A**) The PI3K/pAkt/mTOR pathway regulates brain insulin signaling and glucose metabolism. Glu: Glucose; GLUT: Glucose transporter. (**B**) SGLT-2i ameliorates cerebrovascular dysfunction. (**C**) SGLT-2i mitigates neuroinflammation by suppressing microglial activation and polarization. (**D**) SGLT-2i alleviates oxidative stress and mitochondrial dysfunction. ROS: Oxygen species. (**E**) SGLT-2i influences the pathology of Alzheimer's disease. Aβ: amyloid-beta. (**F**) SGLT-2i promotes the secretion of neurotrophic factors by neurons and astrocytes. BDNF: brain-derived neurotrophic factor; GDNF: glial-derived neurotrophic factor; NT-3/4: neurotrophin-3/4; NGF: nerve growth factor.

**Table 1:** Summary of clinical studies on SGLT-2i in improving DM related cognitive dysfunction.

**Study Type**	**Author (Year)**	**Study Population (Sample Size)**	**Drug/Intervention**	**Follow-up Duration**	**Assessment Methods / Key Metrics**	**Main Results**	**Limitations**
Meta-analyses	Youn *et al* [60] (2024)	T2DM Patient (n=405089)	SGLT-2i (dapagliflozin, empagliflozin, canagliflozin, and ertugliflozin)	1 month to 7.2 years	MMSE, MoCA, RBANS, the verbal fluency test, the Babcock Story Recall Test, and the Attentive Matrices Test	SGLT-2i significantly reduced the risk of dementia (HR=0.68); SGLT-2i significantly improved cognitive function scores (SMD=0.88) in patients with dementia and MCI.	The analysis of dementia incidence risk was based on a limited number of studies and showed substantial heterogeneity (I^2^ = 87%).
	Tian *et al* [61] (2023)	T2DM Patient (n=1590757)	SGLT-2i, GLP-1 RA, thiazolidinedione, DPP-4i, sulfonylurea, and meglitinide	2 years to 15 years	ICD-9-CM, ICD-10, MMSE, MoCA, DSM-IV, and DSM-5	SGLT-2 inhibitors demonstrated the strongest effectiveness in delaying cognitive impairment (SUCRA: 94.3%), dementia (SUCRA: 94.4%), and AD (SUCRA=94.0%) outcomes in patients with T2DM.	The evidence was based on indirect comparisons among hypoglycemic agents, which increased uncertainty regarding their relative effectiveness.
RCTs	Mone *et al* [62] (2024)	Frail elders with DM and CKD (n=119)	Empagliflozin (10 mg/Day)	6 months	MoCA and GST	Empagliflozin significantly improved MoCA and GST scores (*p*<0.05).	This study was conducted at a single center with a small sample size and did not assess the relationship between changes in renal function and cognitive function.
	Zhao *et al* [63] (2022)	Patient with T2DM complicated with MCI (n=96)	Dapagliflozin combined with cognitive-behavioral training (start at 5mg, transition to 10mg)	6 months	C-DMSES, ADL, MMSE, and QOL-AD	Dapagliflozin combined with cognitive-behavioral training significantly improved C-DMSES, ADL, MMSE, and QOL-AD scores (*p*<0.05).	This study was conducted at a single center with a small sample size and did not include a dapagliflozin monotherapy group, limiting the assessment of its independent effects.
Prospective cohort studies	Low *et al* [64] (2022)	Patient with T2DM (n=476)	SGLT-2i (dapagliflozin, empagliflozin, canagliflozin)	6.4 years	12 subsets of cognition tests of RBANS	Long-term use of SGLT2 inhibitors (≥3 years) is significantly positively correlated with RBANS score increase globally and in the language domain.	The study did not analyze residual confounding from known risk factors for cognitive decline, such as mentally stimulating activities, vascular disease, and psychotropic drugs.
	Zhang *et al* [65] (2024)	Patient with T2DM complicated with cognitive impairment (n=290)	Henagliflozin	6 months	MoCA and plasma p-tua181	Henagliflozin treatment was independently associated with improvements in MoCA scores (OR=3.670) and plasma phosphorylated tau181 levels (OR=3.670).	The relatively short 6- month follow-up limits the ability to assess the long-term cognitive effects of SGLT-2i.
	Magurno *et al* [66] (2024)	Patient with T2DM complicated with HFpEF (n=150)	SGLT-2i class	6 months	MMSE, MoCA, GDS, and SPPB	SGLT-2i significantly improved MMSE, MoCA, and SPPB scores, while significantly reducing GDS scores (*p*<0.05).	This single-arm, pre- post design without a control group makes it difficult to rule out confounding effects from disease progression or concurrent treatments.
	Mone *et al* [67] (2022)	Weak elderly patient with T2DM complicated with HFpEF(n=162)	Empagliflozin *vs.* Metformin *vs.* insulin	1 month	MoCA	Empagliflozin significantly improved the MoCA score (OR=3.609).	The one-month duration of this study is relatively short, which limits the assessment of long-term efficacy and safety outcomes.
Retrospective cohort studies	Wu *et al* [68] (2023)	Patient receiving SGLT-2i or DPP-4i (n=106903)	SGLT-2i *vs.* DPP-4i	2.80 years	Dementia diagnosis	Compared to DPP-4i, SGLT-2i reduces the risk of dementia (HR=0.80); dapagliflozin had the best effect (HR=0.67), followed by empagliflozin (HR=0.78).	This study did not report baseline measures like HbA1c, BMI, and renal function, leaving room for residual confounding that may limit interpretation.
	Siao *et al* [69] (2022)	Newly diagnosed T2DM patient (n=206494)	SGLT-2i (dapagliflozin, empagliflozin, and canagliflozin)	2.7 years	Dementia diagnosis	SGLT-2i reduces the risk of new onset dementia (HR: 0.89).	The study was unable to obtain information on adherence to SGLT-2i, which undermines the validity of the outcomes observed.
	Abdullah *et al* [70] (2025)	Patient with T2DM receiving SGLT-2i or DPP-4i (n=118006)	SGLT-2i *vs.* DPP-4i	1.54 years for SGLT-2i; 1.79 years for DPP-4i	Dementia or MCI diagnosis	Compared to DPP-4i, SGLT-2i reduces the risk of dementia (HR=0.78) and MCI (HR=0.86).	The relatively short median follow-up in this study was insufficient to capture the long-term effects of SGLT-2i and DPP-4i.
	Proietti *et al* [71] (2023)	Patient with T2DM accompanied by atrial fibrillation (n=89356)	SGLT-2i (dapagliflozin, empagliflozin, and canagliflozin)	3 years	Dementia diagnosis	Patients who did not receive SGLT-2i treatment had a higher risk of developing new dementia compared to SGLT-2i users (HR=1.66).	The lack of data on the specific dosage and duration of SGLT-2i prevents a meaningful comparison of efficacy differences among SGLT-2i regimens.
	Mui *et al* [72] (2021)	Patient with T2DM receiving SGLT-2i or DPP-4i (n=51460)	SGLT-2i *vs.* DPP-4i	472 days	Dementia, Parkinson's disease, or AD diagnosis	SGLT-2i reduces the risk of new onset dementia (HR=0.41) and new onset Parkinson's disease (HR=0.28).	The relatively short follow-up in this study was insufficient to capture the long-term effects of SGLT-2i and DPP-4i.
Case-control studies	Wium-Andersen *et al* [73] (2019)	Patient with T2DM (n=170417)	SGLT-2i	7.2 years	Dementia diagnosis	SGLT-2i significantly reduced the dementia rate (OR=0.58) and showed a negative correlation with dosage.	In this nested case-control study, due to the recent market introduction of SGLT2 inhibitors, data on long-term patient exposure were limited.
	Ding *et al* [74] (2023)	Middle-aged and elderly patients with T2DM (n=222)	SGLT-2i	16.1 months for the MCI group; 16.4 months for the Control group	MoCA and MMSE	SGLT-2i reduces the risk of MCI (OR=0.337).	The study was a single-center retrospective design with a small sample size, which limited the statistical power of the analyses.

**Abbreviation:** AD: Alzheimer's disease; ADL: Activities of daily living; C-DMSES: Chinese version of the Diabetes Management Self-Efficacy Scale; CKD: Chronic kidney disease; DPP-4i: Dipeptidyl peptidase-4 inhibitors; DSM: Diagnostic and Statistical Manual of Mental Disorders; GDS: Geriatric Depression Scale; GST: 5-meter gait speed test; HFpEF: Heart failure with preserved ejection fraction; HR: Hazard ratio; MCI: Mild cognitive impairment; MMSE: Mini-Mental State Examination; MoCA: Montreal Cognitive Assessment; OR: Odds ratio; QOL-AD: Quality of life in Alzheimer's Disease; RBANS: Repeatable Battery for the Assessment of Neuropsychological Status; RCT: Randomized controlled trial; SGLT-2i: Sodium-glucose cotransporter-2 inhibitors; SMD: Standardized mean difference; SPPB: Short Physical Performance Battery; SUCRA: Surface under the cumulative ranking curve; T2DM: Type 2 diabetes mellitus.

## LIST OF ABBREVIATIONS

ACh: Acetylcholine
AChE: Acetylcholinesterase
AD: Alzheimer's Disease
Aβ: Amyloid-Beta
AGEs: Advanced Glycation End Products
ASL: Arterial Spin Labeling
BACE1: β-Secretase 1
BBB: Blood-Brain Barrier
BDNF: Brain-Derived Neurotrophic Factor
CKD: Chronic Kidney Disease
CNS: Central Nervous System
CREB: cAMP Response Element-Binding Protein
CSD: Chronic Social Defeat
DM: Diabetes Mellitus
DPP-4i: Dipeptidyl Peptidase-4 Inhibitors
DOX: Doxorubicin
GDNF: Glial-Derived Neurotrophic Factor
GLUT: Glucose Transporter
GSK3β: Glycogen Synthase Kinase-3β
HFrEF: Heart Failure With Reduced Ejection Fraction
HFD: High-Fat Diet
HPA: Hypothalamic-Pituitary-Adrenal
I/R: Ischemia/Reperfusion
IDE: Insulin-Degrading Enzyme
IL: Interleukin
IR: Insulin Resistance
LDH-A: Lactate Dehydrogenase A
LTD: Long-Term Depression
MCI: Mild Cognitive Impairment
MMSE: Mini-Mental State Examination
MoCA: Montreal Cognitive Assessment
MR: Myogenic Responses
MRI: Magnetic Resonance Imaging
Npas4: Neuronal PAS Domain Protein 4
NT-3/NT-4: Neurotrophin-3 and Neurotrophin-4
NVU: Neurovascular Unit
OS: Oxidative Stress
PD: Parkinson’s Disease
PDK-1: Pyruvate Dehydrogenase Kinase 1
p-tau181: Phosphorylated Tau at Threonine 181
RCTs: Randomized Controlled Trials
ROS: Reactive Oxygen Species
SGLT-2i: Sodium-Glucose Cotransporter 2 Inhibitors
STZ: Streptozotocin
SVD: Small Vessel Disease
T2DM: Type 2 Diabetes Mellitus
TNF: Tumor Necrosis Factor
VSMCs: Vascular Smooth Muscle Cells
WMH: White Matter Hyperintensities
